# Ingrowing Nails

**Published:** 1878-02

**Authors:** 


### IN-GROWING TOE NAILS

are a source of excessive discomfort and sometimes of almost insufferable pain; formerly the savage mode of treatment was to take a pair of pincers and drag the whole nail out, but now a prompt and painless cure may be effected simply by inserting the dry ses-qui-chloride of iron between the nail and the flesh and powdering the latter with it also, then apply a dry bandage, and a cure follows after two or three applications, a day or two apart.

